# P-1226. Dose Optimization of β-lactams against Carbapenem-Resistant Pseudomonas from Turkiye Defined as Susceptible to Ceftazidime, Cefepime or Piperacillin/Tazobactam

**DOI:** 10.1093/ofid/ofae631.1408

**Published:** 2025-01-29

**Authors:** Ecem Buyukyanbolu, Christian M Gill, Leyla Genc, Mehmet Karakus, Fusun Comert, Barış Otlu, Elif Aktas, David P Nicolau

**Affiliations:** Hartford Hospital, Hartford, Connecticut; Hartford Hospital, Hartford, Connecticut; Health Sciences University Hamidiye Etfal Training and Research Hospital, Department of Medical Microbiology, Istanbul, Istanbul, Turkey; Health Sciences University, Department of Medical Microbiology, Istanbul, Istanbul, Turkey; Bulent Ecevit University, Faculty of Medicine, Department of Medical Microbiology, Zonguldak, Zonguldak, Turkey; Malatya İnönü University, Malatya, Malatya, Turkey; Health Sciences University Hamidiye Etfal Training and Research Hospital, Department of Medical Microbiology, Istanbul, Istanbul, Turkey; Hartford Hospital, Hartford, Connecticut

## Abstract

**Background:**

Due to the increasing prevalence of carbapenem-Resistant *Pseudomonas aeruginosa* (CRPA), effective agents are needed to treat these serious infections. Although CRPA may test susceptible to other β-lactams such as ceftazidime (CAZ), cefepime (FEP), and piperacillin/tazobactam (TZP), reduced potency has been observed amongst CRPA. In this study, we used PKPD analysis to assess the adequacy of the EUCAST Susceptible (S) or Susceptible Increased Exposure (SIE)/(I) doses for CAZ, FEP, and TZP against CRPA clinical isolates.

**Figure 1.**
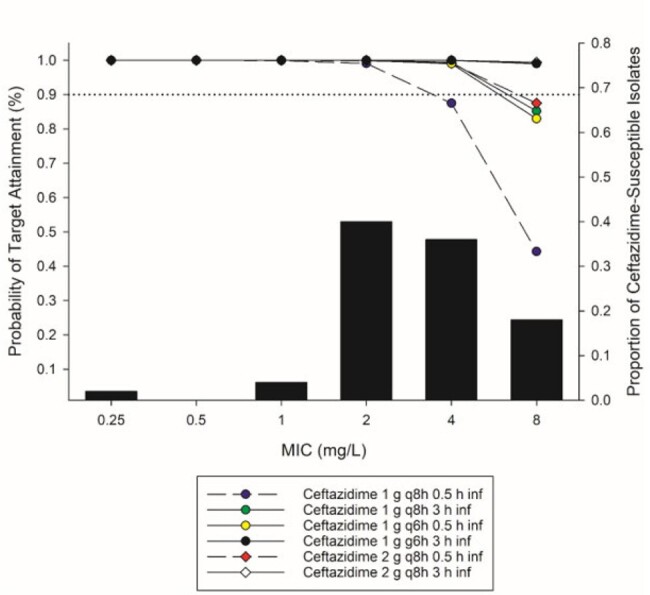
Probability of target attainment (PTA) of various ceftazidime different dosing regimens and the MIC distribution of carbapenem-resistant Pseudomonas aeruginosa (CR-PA).

**Methods:**

CRPA isolates were collected from patients at three Turkish hospitals between January 2017-December 2021. CAZ, FEP, and TZP MICs were determined using broth microdilution according to CLSI methodology. Monte Carlo simulations were performed to determine the probability of target attainment (PTA) for a free time above the MIC (*f*T >MIC) targets for various doses of each agent against isolates defined as susceptible. *f*T >MIC targets were 70% for CAZ or FEP and 50% for TZP. Cumulative fraction of response (CFR) was calculated by integrating PTA with the MIC distribution of the isolates. Optimal PTA and CFR were defined as 90% target achievement.

**Figure 2.**
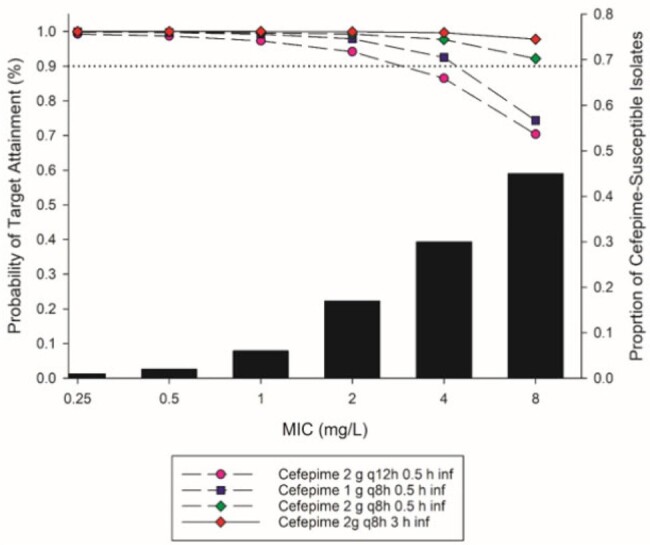
Probability of target attainment (PTA) of various cefepime different dosing regimens and the MIC distribution of carbapenem-resistant Pseudomonas aeruginosa (CR-PA).

**Results:**

In the setting of CR-PA, the percentages of isolates susceptible to CAZ, FEP, and TZP were 49,8%, 47%, and 31,8%, respectively. Reduced potency was noted with 54,1% of CAZ-S isolates had MICs of 4 or 8 mg/L. Of the FEP and TZP-S isolates, MICs at the breakpoint (8 and 16 mg/L, respectively) were the mode with 45,2 and 53,9% of isolates for each, respectively. At an MIC of 8 mg/L for CAZ, the EUCAST standard dose was found to be insufficient with a CFR of 85%. 3-hour infusions of EUCAST SIE doses were required for 90% PTA at MIC of 8 mg/L and an optimized CFR of 100% (Figure 1). For FEP, the SIE dose of 2 g q8h 0.5 h infusion of was effective (CFR 96%), utilization of an extended 3h infusion further optimized the PTA at 8 mg/L (CFR 99%) (Figure 2).

For TZP, the standard dose of 4.5 q6h administered as a 0.5h infusion was inadequate (CFR 86%). A standard TZP dose with an extended infusion (4.5 g IV q8h over 4 h) and the SIE dose 4.5 g IV q6h 3 h inf resulted in CFRs >95% (Figure 3).

**Figure 3.**
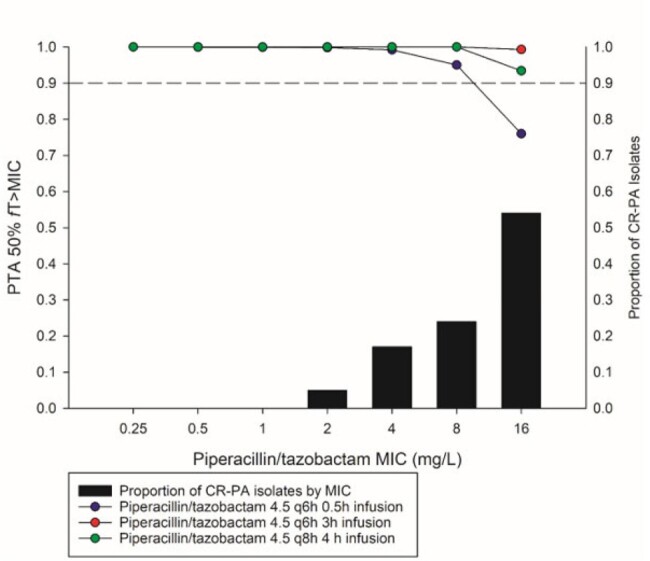
Probability of target attainment (PTA) of various piperacillin/tazobactam different dosing regimens and the MIC distribution of carbapenem-resistant Pseudomonas aeruginosa (CR-PA).

**Conclusion:**

These data support the EUCAST SIE breakpoints for FEP and TZP. To optimize PTA at the SIE breakpoint for CAZ, prolonged infusion is required.

**Disclosures:**

**Christian M. Gill, PharmD**, Cepheid: Grant/Research Support|Entasis: Grant/Research Support|Everest Medicines: Grant/Research Support|Shionogi: Grant/Research Support **David P. Nicolau, PharmD**, CARB-X: Grant/Research Support|Innoviva: Grant/Research Support|Innoviva: Honoraria|Merck: Advisor/Consultant|Merck: Grant/Research Support|Merck: Honoraria|Pfizer: Advisor/Consultant|Pfizer: Grant/Research Support|Pfizer: Honoraria|Shionogi: Advisor/Consultant|Shionogi: Grant/Research Support|Shionogi: Honoraria|Venatorx: Grant/Research Support

